# Effect of Natalizumab Treatment on Circulating Plasmacytoid Dendritic Cells: A Cross-Sectional Observational Study in Patients with Multiple Sclerosis

**DOI:** 10.1371/journal.pone.0103716

**Published:** 2014-07-30

**Authors:** Pia Kivisäkk, Katiana Francois, Julvet Mbianda, Roopali Gandhi, Howard L. Weiner, Samia J. Khoury

**Affiliations:** 1 Center for Neurologic Diseases, Brigham and Women’s Hospital, Harvard Medical School, Boston, Massachusetts, United States of America; 2 Partners Multiple Sclerosis Center, Department of Neurology, Brigham and Women’s Hospital, Harvard Medical School, Boston, Massachusetts, United States of America; 3 Abu Haidar Neuroscience Institute, American University of Beirut, Beirut, Lebanon; Beth Israel Deaconess Medical Center, Harvard Medical School, United States of America

## Abstract

**Objectives:**

Dendritic cells (DCs) serve a critical role both in promoting and inhibiting adaptive immunity. The goal of this study was to investigate the effect of natalizumab (NTZ) treatment on DC numbers, phenotype, and function in patients with multiple sclerosis (MS).

**Methods:**

Frequency and phenotype of myeloid and plasmacytoid DCs (MDCs and PDCs, respectively) were analyzed in blood from two separate cohorts of untreated, interferon-treated, or NTZ-treated MS patients. In addition, PDCs were stimulated with CpG-containing oligonucleotides or co-cultured with homologous T cells in the presence or absence of NTZ in vitro to determine functional effects of NTZ treatment.

**Results:**

We observed that NTZ treatment was associated with a 25–50% reduction in PDC frequency in peripheral blood as compared to untreated MS patients, while the frequency of MDCs was unchanged. PDCs in NTZ-treated patients displayed a mature, activated phenotype with increased expression of HLA-DR, TLR9, CCR7, IL-6 and IL-12. In contrast, in vitro treatment with NTZ did not increase markers of PDC activation or their ability to induce T cell differentiation.

**Conclusion:**

Our study shows that NTZ treatment is associated with a reduced frequency of PDCs in the peripheral circulation, but that PDCs in NTZ-treated individuals display an activated phenotype. Taken together the data suggests that transmigration of activated PDCs is preferentially affected by blockade of integrin α4 leading to an increased frequency of activated PDCs in blood.

## Introduction

Natalizumab (NTZ) is a humanized monoclonal antibody (mAb) against the α4 subunit (CD49d) of the α4β1 (VLA-4) and α4β7 integrins that has been approved for the treatment of multiple sclerosis (MS) due to its ability to reduce disease activity and severity in patients with relapsing-remitting MS (RRMS) [1]. NTZ is known to inhibit T cell trafficking to the CNS by blocking the interactions between VLA-4 on T cells and VCAM-1 on cerebral endothelial cells, a step that is required for T cell extravasation to the CNS. Previous studies have shown that NTZ treatment in MS is associated with increased frequencies of activated CD4+ T cells producing proinflammatory cytokines such as IFN-γ, TNF, and IL-17 in peripheral blood [2]–[5]. Such effects of NTZ are, however, not limited to activated CD4+ T cells, but an increase in peripheral blood frequencies of total T cells, B cells, and NK cells has also been demonstrated, while frequencies of monocytes were decreased [6]–[9]. In addition to sequestration of activated cells in the peripheral circulation, these changes in immune cell composition are believed to be mediated by a higher release of VLA-4+ hematopoetic precursor cells from the bone marrow and a decreased retention of memory and marginal zone B cells within secondary lymphoid organs [3], [8], [10].

Dendritic cells (DCs) serve a critical role both in promoting and inhibiting adaptive immunity, depending on the DC subset and maturation state. Cytokines produced by DCs influence the differentiation of effector T helper cells into distinct subsets with unique functions, such as Th1, Th2, Th17, Tr1 or Treg subsets [11]. Integrin α4 is expressed by DCs [12] and preliminary studies indicate that NTZ treatment in MS is associated with changes in DC trafficking as evidenced by a potential reduction in the frequency of circulating plasmacytoid DCs (PDCs) and in the number of CD209+ DCs in the perivascular spaces of the brain [13], [14]. Antigen-presenting cells (including DCs) in the cerebral perivascular spaces are believed to be continuously replaced by cells migrating from peripheral blood and it is plausible that the observed reduction in DC numbers is due to impaired migration of DCs from peripheral blood [15], [16].

The goal of this study was to investigate the effect of NTZ treatment on the frequency of circulating DCs, their phenotype, and ability to induce T cell differentiation or polarization in patients with MS. Our data showed that NTZ treatment is associated with reduced frequencies of circulating PDCs and that PDCs in NTZ treated individuals display an activated phenotype.

## Methods

### Patients

For our initial studies, we obtained blood from 25 patients with RRMS treated with NTZ for >1 year (Tysabri, Biogen Idec, Weston, MA; 300 mg IV every 4 weeks). Twenty-five RRMS patients treated with IFN-β (Avonex, Biogen Idec; Betaseron, Bayer Healthcare, Wayne, NJ; Rebif, EMD Serono, Rockland, MA; all at recommended doses) for >1 year and 25 untreated RRMS patients were included as controls (cohort 1; Table 1). Later, we obtained blood from 56 additional RRMS patients to confirm our findings (cohort 2). Of these, 19 were treated with NTZ, 16 were treated with IFN-β, and 21 patients were untreated. Blood from the NTZ-treated patients in cohort 1 was obtained immediately before an infusion to reflect serum trough levels, while blood from patients in cohort 2 was obtained at regular clinical visits regardless of the timing of their NTZ infusions. Untreated patients had been without any immunomodulatory treatment for ≥3 months and had never received any immunosuppressive or immunomodulatory medications other than corticosteroids, IFN-β, or glatiramer acetate. None of the patients treated with NTZ or IFN-β had developed neutralizing antibodies against the drug. Blood for in vitro experiments was obtained from 12 healthy donors (5 women, 7 men; mean age: 30.9 years, range 22–50). The study was approved by the Institutional Review Board at Brigham and Women’s Hospital (protocol numbers: 2009P002380 and 2001P001431) and all subjects provided written informed consent.

**Table 1:** Patient demographics.

	Untreated	Tysabri	Interferon	p-value
**Cohort I**				
Number of donors	25 (4M/21F)	25 (9M/16F)	25 (6M/19F)	*p* = 0.19
Age (years)	41.8 (22–70)	39.1 (23–45)	45.3 (24–65)	*p* = 0.15
Disease duration (years)	8.4 (0–33)	9.7 (3–38)	9.0 (2–26)	*p* = 0.81
EDSS	1.3 (0–3)	1.8 (0–6.5)	1.6 (0–6)	*p* = 0.78
Treatment duration (months)	n/a	27.5 (14–42)	70.9 (15–147)	n.d.
Relapses within 24 months*	4/14	3/18	5/16	*p* = 0.68
Time to relapse (months)	7.0 (1–20)	12.0 (3–24)	10.4 (1–23)	n.d.
**Cohort II**				
Number of donors	21 (1M/20F)	19 (3M/16F)	16 (3M/13F)	*p* = 0.33
Age (years)	43.4 (22–64)	42.9 (26–56)	46.8 (30–62)	*p* = 0.45
Disease duration (years)	9.8 (0–20)	8.8 (2–22)	9.4 (4–18)	*p* = 0.82
EDSS	1.1 (0–6)	1.7 (0–6)	1.6 (0–3)	*p* = 0.11
Treatment duration (months)	n/a	31.4 (5–55)	54.4 (5–127)	n.d.

*Number of donors with a documented relapse during 24 months of follow-up. M = male; F = female. Data is expressed as mean (range) unless otherwise specified. EDSS = Expanded Disability Status Scale. Differences between the groups were determined using ANOVA (age and disease duration), Kruskal-Wallis one-way analysis of variance (EDSS), or Fisher exact test (gender and presence of relapses; p-value shows comparison Untreated vs. Tysabri).

### Isolation of peripheral blood mononuclear cells (PBMCs)

Peripheral blood mononuclear cells (PBMCs) were isolated through density centrifugation on Ficoll-Paque (GE Healthcare, Pittsburgh, PA) within two hours of blood collection and used either fresh or after being cryopreserved in liquid nitrogen according to the protocols of the Immune Tolerance Network (www.immunetolerance.org). Frozen samples were thawed and analyzed in batches containing equal numbers of untreated, NTZ- and IFN-β-treated patients to reduce batch-to-batch effects. A quality control sample consisting of PBMCs isolated from a buffy coat was included in each batch to detect batch-to-batch variability. Batches with too high variability were excluded from further analysis.

### Frequencies of DCs and their phenotype and gene expression

Frozen PBMCs were used to determine the frequency of DC subsets and their expression of cell surface molecules using flow cytometry. DCs were defined as being negative for hematopoietic lineage markers (CD3, CD14, CD16, CD19, CD20 and CD56; Lineage Cocktail 1 (Lin), BD Biosciences, San Jose, CA) and positive for CD11c (myeloid DCs, MDCs) or CD123 (PDCs). In addition, all DCs expressed HLA-DR at high levels. Dead cells were excluded by a viability staining (LIVE/DEAD Fixable Dead Cell Stain, Invitrogen). Intracellular staining was performed after fixation and permeabilization (BD Cytofix/Cytoperm, BD Biosciences). Cells were acquired on a BD LSR II flow cytometer (BD Biosciences) and analyzed using FlowJo (Tree Star, Ashland, OR). Isotype control antibodies were used to determine background fluorescence levels.

PDCs and MDCs for gene expression analysis were isolated from PBMCs using a FACSAria cell sorter (BD Biosciences) using the same definition as above and were >95% pure. The separated cells were lysed using RLT buffer (Qiagen, Valencia, CA) and frozen at −80°C.

### Antibodies for flow cytometry

CD4 APC (clone RPA-T4), CD11c APC (B-ly6), CD40 PE (5C3), CD45 PE (H130), CD49d PE-Cy5 (9F10), CD80 PE (L307.4), CD83 PE (HB15e), CCR7 PE (150503), Bcl-2 PE (Bcl-2/100), TLR9 PE (eB72-1665), HLA-DR PE-Cy5 (G46-6), Lineage Cocktail-1 FITC, and 7-AAD were obtained from BD Biosciences. CD86 PE-Cy5 (It2.2), and CD123 PE-Cy7 (6H6) were obtained from BioLegend (San Diego, CA). Polyclonal TLR7 PE was obtained from Imgenex (San Diego, CA). Bcl-xL PE (H5) was obtained from St Cruz (Dallas, TX).

### *In vitro* experiments

Purified PDCs isolated from freshly isolated PBMCs were stimulated with ODN-2006 or −2336 (both at 5 µM; InvivoGen, San Diego, CA; 10,000 cells/well) for 4 hours in the presence of 15 µg/ml of NTZ or IgG4 (Sigma) after which cells were lysed using RLT buffer and frozen at −80°C. Freshly isolated PBMCs were stimulated with ODN-2006 or -2336 (both at 5 µM; InvivoGen; 500,000 cells/well) for 18 hours in the presence of 15 µg/ml of NTZ or IgG4 (Sigma Aldrich, St Louis, MO). Cells were retrieved and stained for flow cytometric analysis.

For co-culture experiments, PDCs were isolated from freshly isolated PBMCs by negative selection using magnetic beads (Plasmacytoid Dendritic Cell Isolation Kit II, Miltenyi, Auburn, CA). More than 90% of separated cells were Lin−/CD123+/CD11c−/HLA-DR+. Cells were stimulated with 5 µM ODN-2006 for 18 hours (5,000 cells/well). CD4+ T cells were isolated from homologous blood kept at +4°C overnight by negative selection using magnetic beads (CD4+ T Cell Isolation Kit II, Miltenyi) and added to each well (20,000 cells/well). Co-cultures were incubated for five days in the presence of anti-CD3 (HIT3a, 1 µg/ml, BD Biosciences) and 20U IL-2. Live CD4+ T cells were isolated by FACS sorting and lysed using RLT buffer.

### RNA isolation and real-time PCR

RNA was isolated from frozen cell lysates using Qiagen RNeasy micro kit (Qiagen). Total RNA was converted to complementary DNA using Taqman reverse transcription reagents (Applied Biosystems, Carlsbad, CA). Quantitative PCR was performed using ViiA 7 Real-Time PCR System (Applied Biosystems). All primers and probes were obtained from Applied Biosystems and used according to standard protocols. A comparative threshold cycle (CT) value was normalized for each sample using the following formula: ΔCT = CT_(gene of interest)_-CT_(B2M)_, and the relative expression calculated using the formula 2^−ΔCT^.

### Statistical analysis

Comparisons between NTZ-treated patients and the two control groups (IFN-treated patients and HC) in all ex vivo experiments were performed using the non-parametric Mann-Whitney U-test. MFI values were normalized to the geometrical mean of each batch (n = 5) that contained equal numbers of donors from each treatment group. Effects of NTZ treatment in vitro were determined using a paired t-test comparing NTZ- and isotype-treated cultures from individual donors. The relation between treatment duration and PDC frequency was assessed using linear regression. All p-values are two-tailed and differences were considered statistically significant when p<0.05. Given the exploratory nature of this study, no corrections for multiple comparisons were completed. Differences in demographic characteristics or clinical status between the groups were determined using ANOVA (age and disease duration), Kruskal-Wallis one-way analysis of variance (EDSS), or Fisher exact test (gender and presence of relapses). Statistical analysis was performed using Prism 6 for Mac OS X (GraphPad Software Inc., San Diego, CA).

## Results

### NTZ-treated patients have a decreased frequency of plasmacytoid DCs in peripheral blood

**Figure 1:**
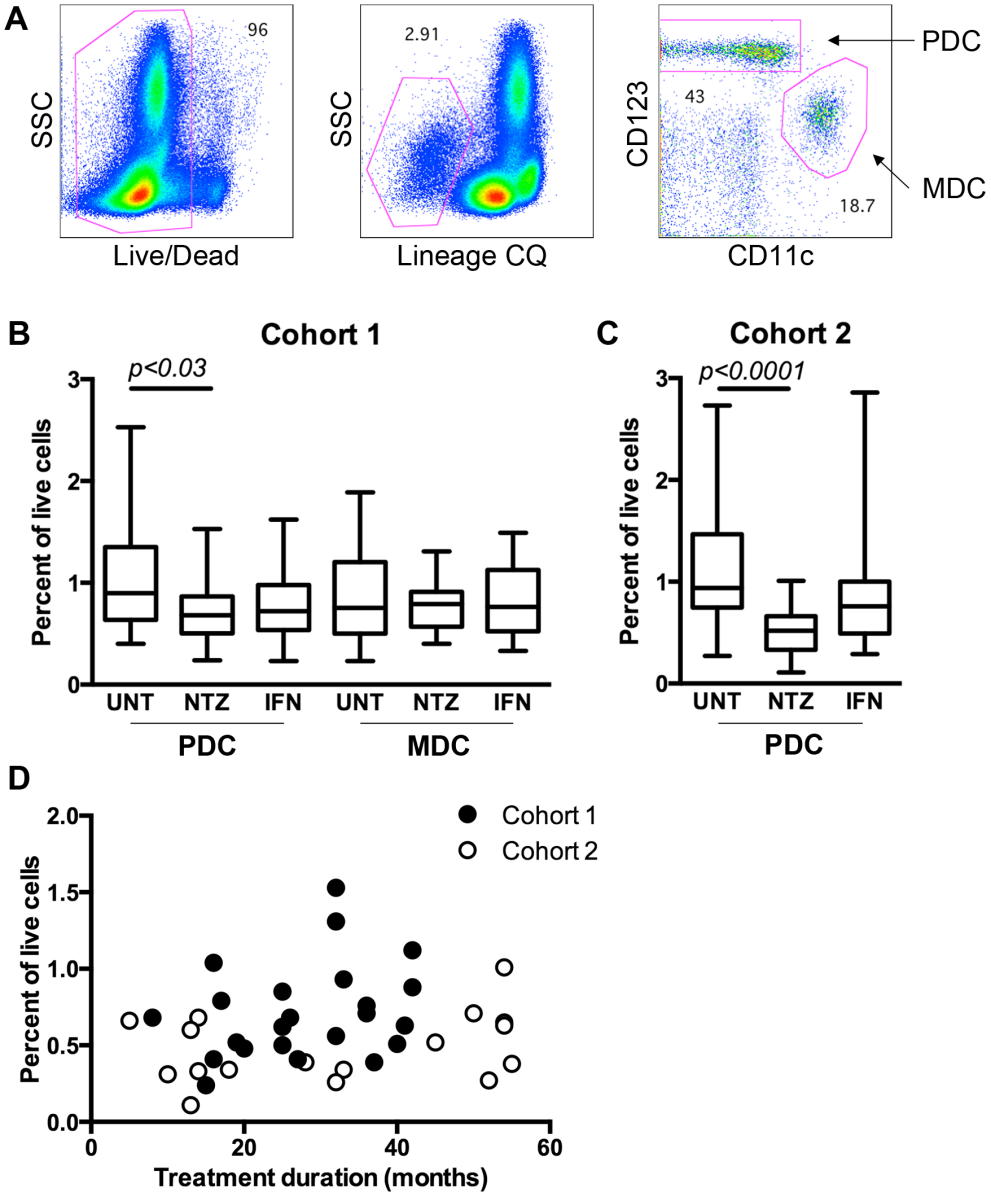
Frequency of myeloid and plasmacytoid dendritic cells in peripheral blood. Frequency of myeloid and plasmacytoid dendritic cells (MDCs and PDCs) was determined in frozen cells from relapsing-remitting MS patients using flow cytometry. A. Dead cells were excluded using a viability dye (LIVE/DEAD), followed by exclusion of cells expressing mature hematopoietic lineages using a Lineage cocktail (CQ). PDCs and MDCs were identified based on their expression of CD123 and CD11c, respectively. B–C. Frequency of MDCs and PDCs in untreated MS patients (UNT) and MS patients treated with natalizumab (NTZ) or interferon-β (IFN) from Cohort 1 (B) and Cohort 2 (C). Graph shows mean, interquartile range, and min-max range. Frequency of PDCs in NTZ-treated patients in relation to duration of treatment in patients from Cohort 1 (filled) or Cohort 2 (open) (D).

Patients treated with NTZ had roughly 25% less PDCs in peripheral blood (0.72±0.30%) compared to untreated patients (1.04±0.53%) in our first cohort of 75 patients (p = 0.03; Fig. 1A–B). In order to confirm the results, the frequency of PDCs was analyzed in a second cohort of 56 patients, which showed an even more pronounced difference between the groups (NTZ: 0.50±0.22%; UNT: 1.08±0.59%; p<0.0001; Fig. 1C). There was no correlation between the frequency of PDCs in NTZ-treated patients and treatment duration, either in the first (p = 0.21, r^2^ = 0.07) or second cohort (p = 0.21, r^2^ = 0.10; Fig. 1D). There was a decrease in the frequency of PDCs in IFN-β-treated patients in the first cohort (0.74±0.33%; p = 0.06), but this was not statistically significant nor reproduced in the second cohort (0.92±0.67%; p = 0.2; Fig. 1B–C). The frequency of MDCs was not affected by NTZ or IFN-β treatment (Fig. 1A–C).

**Figure 2:**
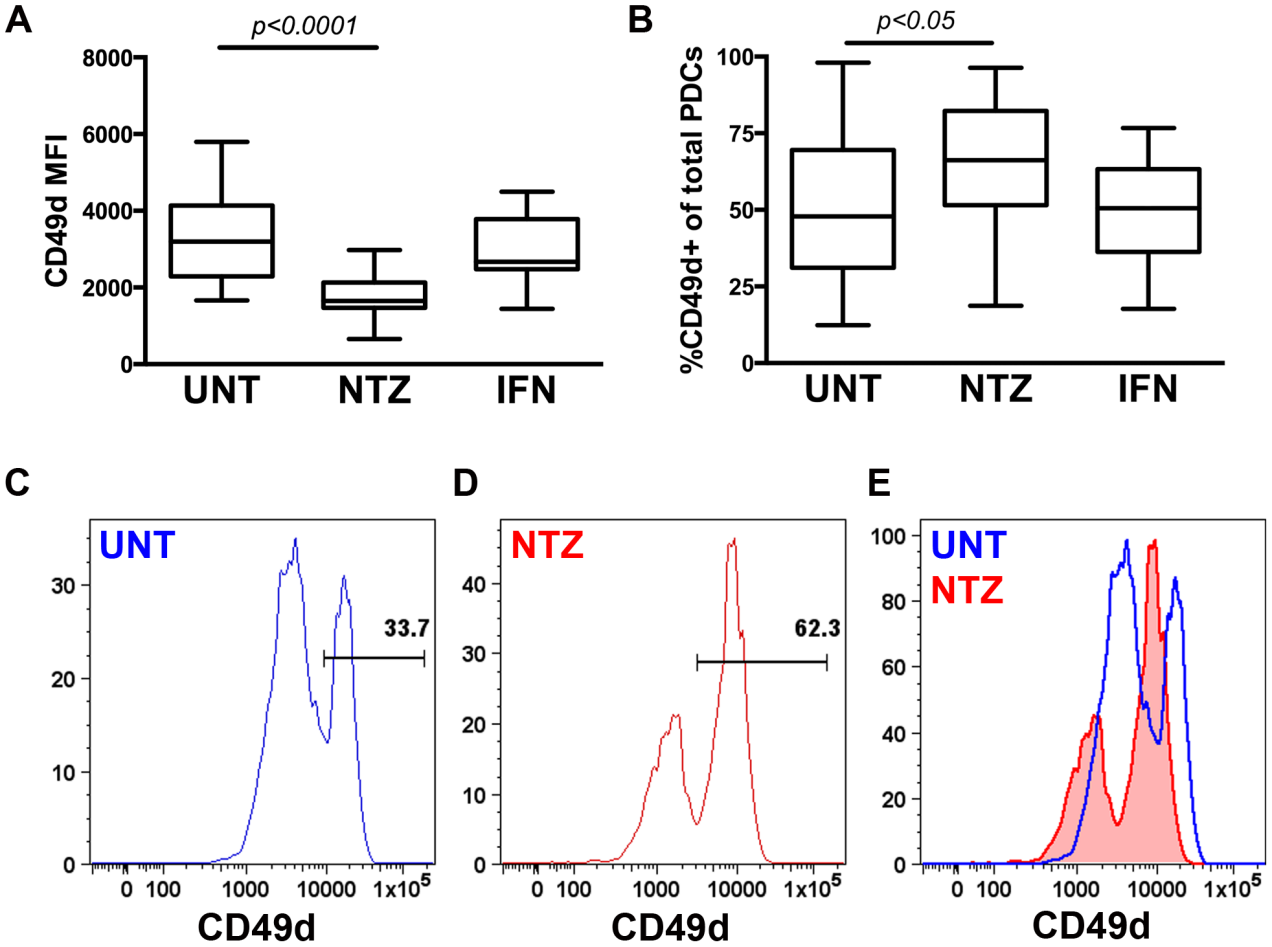
CD49d expression on plasmacytoid dendritic cells. Mean fluorescence intensity (MFI) of CD49d on plasmacytoid dendritic cells (PDCs)(A) and frequency of PDCs expressing CD49d (B), in peripheral blood from untreated MS patients (UNT) and MS patients treated with natalizumab (NTZ) or interferon-β (IFN) determined by flow cytometry. Box-and-whisker plots showing mean, interquartile range, and min-max range. Representative histograms showing CD49d staining on PDCs from an UNT (blue open; C, E) and a NTZ-treated (red filled; D, E) patient.

We confirmed that PDCs express CD49d (integrin α4), a surface antigen on leukocytes which NTZ binds to, using both flow cytometry and rt-PCR. Intriguingly, while we observed that the frequency of CD49d+ PDCs was increased in NTZ-treated patients, it appeared as the intensity of the CD49d staining (MFI) was reduced both in the CD49d^hi^ and CD49d^low^ populations and that the total staining intensity of CD49d was decreased on PDCs from NTZ-treated patients similar to a previous report [14] (Fig. 2A–E).

**Figure 3:**
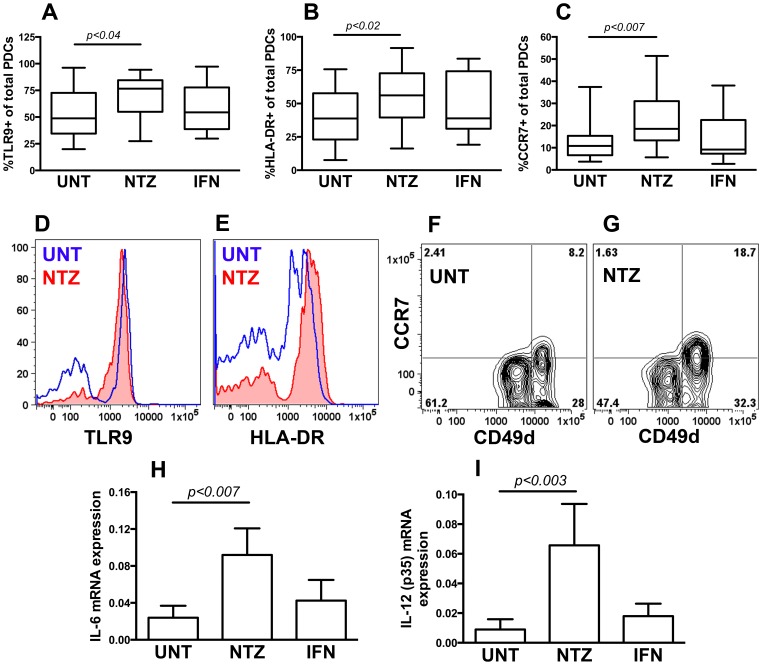
Phenotype and cytokine expression of plasmacytoid dendritic cells. Frequency of plasmacytoid dendritic cells (PDCs) expressing TLR9 (A), HLA-DR (B), and CCR7 (C) in peripheral blood from untreated MS patients (UNT) and MS patients treated with natalizumab (NTZ) or interferon-β (IFN) determined using flow cytometry. Representative histograms showing TLR9 (D) and HLA-DR (E), staining on PDCs from an UNT (blue open) and a NTZ-treated (red filled) patient. Flow plots showing CCR7 staining on CD49d^hi^ and CD49d^low^ PDCs from an UNT (F) and a NTZ-treated (G) patient. Expression of IL-6 (H) and IL-12 (p35) (I) mRNA in PDCs isolated from the patients described in A-C using FACS sorting and rt-PCR without ex-vivo stimulation. Graph shows mean, interquartile range, and min-max range (A–C) or mean and SD (H–I).

PDCs from NTZ-treated patients expressed higher levels of HLA-DR, CCR7 and Toll-like receptor-9 (TLR9), three markers associated with cellular activation, maturation and trafficking abilities in PDCs, compared to untreated patients (Fig. 3A–G), while the expression of the anti-apoptotic Bcl-2 and Bcl-xL were comparable in the two groups (data not shown). PDCs isolated directly *ex vivo* using FACS sorting of blood from NTZ-treated patients expressed significantly higher levels of IL-6 and IL-12 (p35) mRNA compared to PDCs isolated from untreated patients (Fig. 3H–I), while we observed no differences in the expression of IL-1β, IL-10, IL-23, IL-27 or TGF-β mRNA (data not shown). We did not observe any changes in the expression of these markers in PDCs from IFN-β treated patients (Fig. 2, 3); neither was NTZ- or IFN-β treatment associated with changes in the surface phenotype or cytokine expression in MDCs (data not shown).

### *In vitro* treatment with NTZ is associated with a slight decrease in the expression of CD40, CD83, and HLA-DR mRNA in CpG-stimulated PDCs

**Figure 4:**
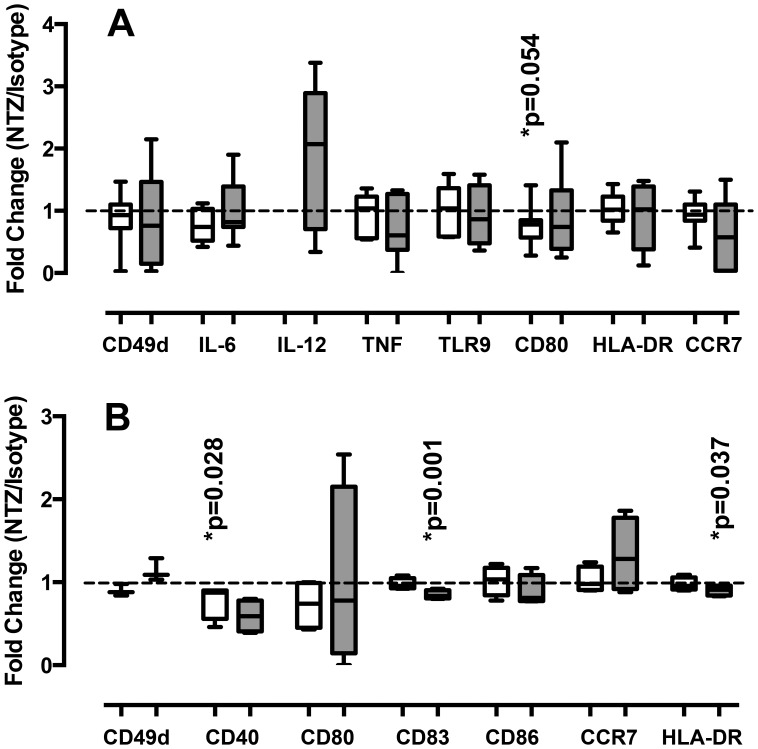
In vitro effects of natalizumab treatment on plasmacytoid dendritic cells. Effects of natalizumab (NTZ) treatment on plasmacytoid dendritic cells (PDCs) stimulated in vitro with CpG-containing oligonucleotides (open: ODN-2006; gray: ODN-2336). (A) PDCs were isolated from healthy controls using FACS sorting and stimulated in the presence of 15 µg/ml of NTZ or an isotype control for 4 hours. The expression of CD49d, IL-6, IL-12 (p35), TNF, TLR9, CD80, HLA-DR, and CCR7 mRNA was determined using rt-PCR. (B) Total PBMCs were stimulated in the presence of 15 µg/ml of NTZ or an isotype control for 18 hours. Surface expression of CD49d, CD40, CD80, CD83, CD86, CCR7, and HLA-DR was determined on PDCs using flow cytometry. Graphs shows fold change in expression comparing cultures treated with NTZ to cultures treated with an isotype control.

PDCs isolated from healthy volunteers were stimulated in vitro with ODN-2006 or -2336, two synthetic CpG-containing oligonucleotides known to stimulate PDCs [17], in the presence or absence of NTZ (15 µg/ml). There was a trend towards reduced expression of CD80 mRNA after NTZ treatment in PDC cultures stimulated with ODN-2006, a type B ODN known to upregulate costimulatory and Ag-presenting molecules on PDCs (NTZ: 3.53±2.45; Isotype: 4.45±2.76; p = 0.054; n = 7) [18]. In vitro treatment with NTZ did not affect CD80 mRNA expression in cultures stimulated with ODN-2336, a type A ODN that induces large amounts of IFN-α by PDCs [19], nor did it result in increased expression of CD49d, HLA-DR, TLR9, CCR7, IL-6, or IL-12 mRNA in PDCs, as observed ex vivo in NTZ-treated MS patients, either when PDCs were stimulated with ODN-2006 or -2336 (Fig. 4A).

It is plausible that the lack of effects of NTZ on PDCs in pure PDC cultures is reflecting that NTZ modulates PDCs indirectly through factors secreted from other cells present in peripheral blood. In order to simulate this, we stimulated total PBMCs with ODN-2006 or -2336 in the presence or absence of NTZ and analyzed surface expression of co-stimulatory molecules, CD49d, CCR7, and HLA-DR in PDCs using flow cytometry. NTZ treatment of PBMCs did not change the frequency or viability of PDCs (data not shown). Contrary to the findings ex vivo, we observed a statistically significant decrease in CD40 expression in ODN-2006 stimulated cultures (NTZ: 23.81±22.24; Isotype: 27.20±23.80; p = 0.028; Fig. 4B) and a trend towards decreased CD40 expression in ODN-2336 stimulated cultures (NTZ: 15.77±20.65; Isotype: 21.64±28.36; p = 0.22; Fig. 4B). In addition, the expression of CD83 and HLA-DR were decreased after NTZ treatment in ODN-2336 stimulated cultures (NTZ: 33.64±18.93; Isotype: 38.74±19.15; p = 0.001; and NTZ: 35.89±18.47; Isotype: 39.18±18.33; p = 0.037; respectively; Fig. 4B).

### NTZ effects on the functional ability of PDCs to stimulate CD4 cell responses

**Figure 5:**
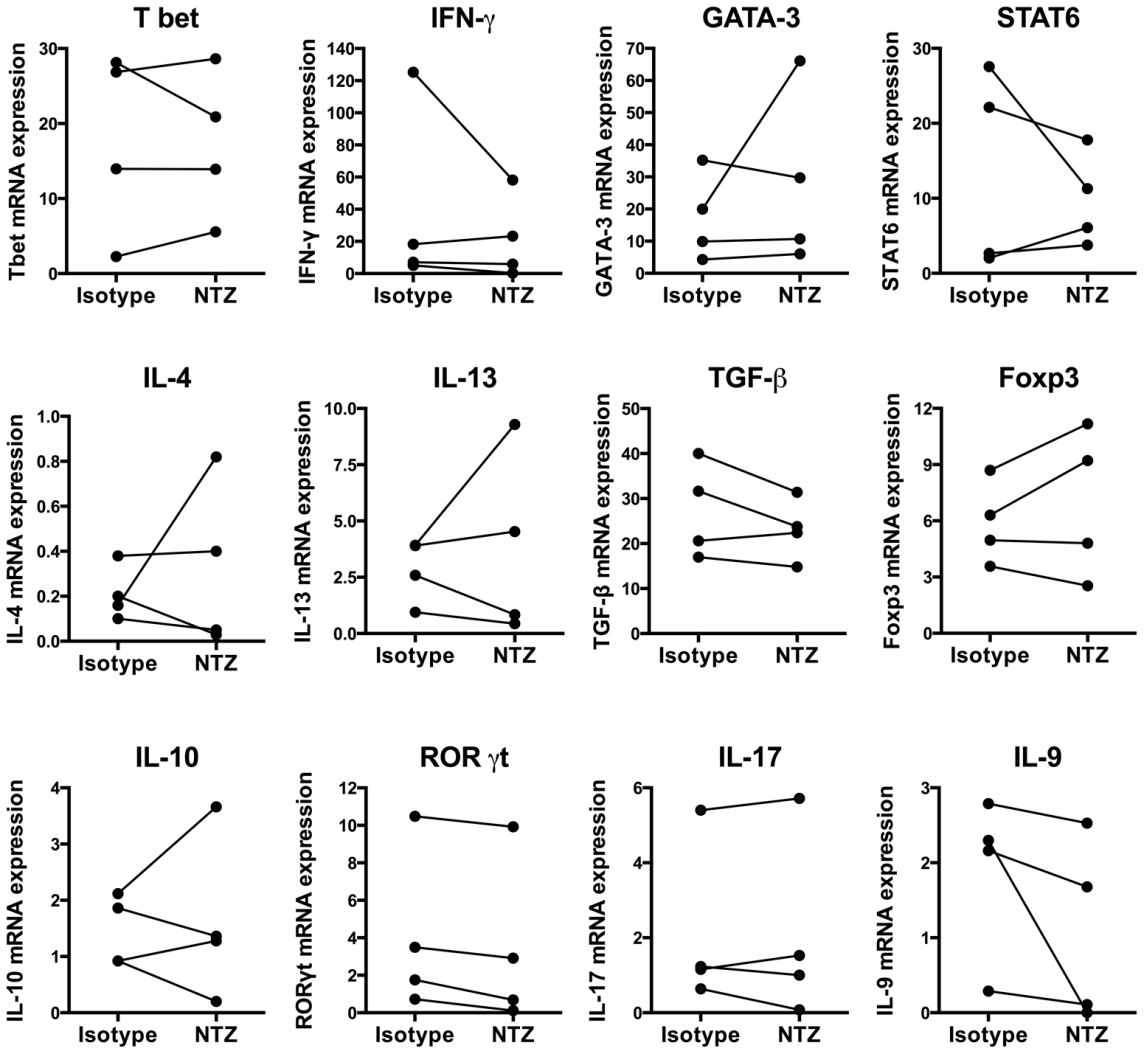
Effects of natalizumab treatment on plasmacytoid dendritic cells function. Effects of natalizumab (NTZ) treatment on the functional ability of plasmacytoid dendritic cells (PDCs) to stimulate CD4 cell responses. PDCs were isolated from healthy controls using FACS sorting and stimulated with ODN-2006 for 18 hours in the presence of 15 µg/ml of NTZ or an isotype control and homologous CD4+ T cells were added. CD4+ T cells were isolated after an additional 6 days of incubation and cytokine mRNA expression determined using rt-PCR. Graph shows expression in cultures treated with NTZ or an isotype.

In order to address if NTZ has any effects on the functional ability of PDCs to stimulate CD4+ T cell responses, we isolated PDCs from healthy volunteers, stimulated them with ODN-2006 in the presence or absence of NTZ, co-cultured them with purified homologous CD4+ T cells, and determined cytokine mRNA expression in CD4+ T cells using rt-PCR. Under the conditions used, we could not demonstrate any changes in the mRNA expression of a panel of cytokines and transcription factors selected to reflect Th1/Th2/Th17/T reg differentiation in CD4+ T cells (IL-4, IL-9, IL-10, IL-13, IL-17, IFN-γ, TGF-β, RORγt, T bet, GATA-3, STAT6, and Foxp3; Fig. 5).

## Discussion

In this study, we observed that the frequency of PDCs was 25–50% lower in blood from NTZ-treated compared to untreated MS patients in two separate cohorts. Interestingly, the decrease seemed to be dose-dependent as a more pronounced decrease was observed in patients analyzed at clinical visits unrelated in time to the infusions than in patients analyzed at the end of a dosing interval, immediately before an infusion. This is consistent with a small study of six patients followed for one year after initiation of NTZ therapy, which previously reported a non-statistically significant decrease in PDCs [14]. We did not see any effect of treatment duration, which ranged from 5–55 months, on PDC frequency. The effects of NTZ on lymphocyte numbers are known to occur within the first months of treatment and remain persistent for treatment durations of 2–3 years [8], [20], [21].

As NTZ treatment is known to increase the numbers of multiple lymphocyte subsets in peripheral blood [6]–[8], [22], we would have expected to find an increased frequency of PDCs in the circulation during NTZ treatment [2]–[4]. We calculated the frequency of PDCs in total live PBMCs and it is possible that the decrease in PDC frequency is reflecting an increase in other lymphocyte subsets during NZT treatment rather than a decrease in PDC numbers per se [22]. MDCs are present at comparable levels to PDCs in peripheral blood but unlike PDCs the frequency of MDCs did not decrease after NTZ treatment. The frequency of MDCs could be considered an internal control for any change in total lymphocyte counts. Myeloid cells express CD49d, but NTZ treatment does not seem to affect numbers and/or frequencies of monocytes or MDCs [6], [7], [14], [21], [23]. Thus, we would suggest that the decrease in PDC frequency in NTZ-treated subjects is specific and not merely related to changes in other cell populations.

The observed reduction in PDC frequency in blood may reflect a general decrease in CNS inflammation following NTZ treatment resulting in reduced PDC mobilization. Resting PDCs can under inflammatory conditions enter peripheral tissues, respond to the local environment, and become activated. Activated PDCs may then acquire antigen and return to secondary lymphoid organs [24]. In contrast to other DC populations, PDCs do not migrate to secondary lymphoid organs through afferent lymphatic vessels, but through the blood stream [25]. Interestingly, we observed that while the frequency of PDCs were decreased in NTZ-treated patients, the cells detected displayed a phenotype consistent with activated, mature PDCs, characterized by increased expression of HLA-DR, TLR9, CCR7, IL-6 and IL-12, which would argue that additional factors besides less CNS inflammation contribute to the changes is PDC frequency and phenotype.

Extravasation of PDCs through high endothelial venules (HEV) has been shown to follow the same multi-step procedure of tethering, rolling, adhesion, and transmigration as has been described for T cells [26]. Blocking experiments have shown that integrins, including both the α4 and the β1 subunits of VLA-4, are involved in the attraction of PDCs to endothelial cells both in secondary lymphoid organs and peripheral tissue [27]–[29]. We observed the seemingly contradictory finding that NTZ-treated patients had a higher frequency of CD49d+ PDCs, but that the staining intensity of each cell was lower, indicating less CD49d molecules on the cell surface. The staining intensity of CD49d has previously been shown to be reduced on most lymphocyte subsets, including PDCs, during NTZ treatment presumably due to internalization and/or shredding [5], [14], [23]. On the other hand, integrin α4 is upregulated on DCs upon maturation [12] consistent with the increased expression of other markers for activated, mature PDCs in NTZ-treated patients in our study. It is possible, although speculative, that mature PDCs with their higher expression of CD49d are more dependent on VLA-4 mediated adhesion than immature PDCs. This could result in relatively more efficient blockade of the migration of activated PDCs to lymphatic tissue compared to the migration of resting PDCs to inflamed tissue, leading to an increased frequency of activated PDCs in the peripheral circulation. Further in vitro experiments with chemotaxis assays would be needed to fully understand differences in trafficking behavior between activated and resting PDCs.

We did not find any evidence indicating that NTZ treatment would result in direct PDC activation during in vitro experiments using PDCs stimulated with synthetic CpG-containing oligonucleotides. In contrast, we observed a slight decrease in the expression of CD40, CD83 and HLA-DR on these cells. This is in agreement with a previous study showing reduced ability of NTZ-treated PDCs to stimulate PPD-specific CD4+ T cell proliferation [14]. It is likely that this discrepancy between in vivo and in vitro effects of NTZ reflects effects of NTZ on PDC trafficking behavior rather than direct effects on the activation or maturation of the cells. Although VLA-4 cross-linking has been associated with tyrosine phosphorylation and T cell costimulation [30], studies on T cells have shown little evidence that NTZ exerts a direct effect on cellular activation or maturation [3]–[5], [31]. This mechanism of action differs from other therapies in MS with effects on PDCs, such as IFN-β treatment, which has been shown to directly affect PDCs during in vitro stimulation resulting in reduced proinflammatory cytokine secretion, reduced expression of CCR7, increased expression of programmed death ligand 1 (PD-L1), and increased IL-10 secretion [32].

PDCs are key regulators in the development of both innate and adaptive immune responses. They have the ability to quickly respond to viral antigens by secreting large amounts of type I interferons [33], but they also upregulate the expression of MHC class II and costimulatory molecules upon maturation, enabling them to engage in antigen presentation and T cell activation [34]. Depending on the localization and the specific microenvironment, PDCs may have both immunogenic and tolerogenic functions [35]. We performed co-culture experiments to determine the functional ability of NTZ-treated PDCs to stimulate CD4 cell responses in vitro. Although the small number of donors analyzed precludes finding any minor differences, our data did not demonstrate any large effects of NTZ on PDCs ability to affect Th1/Th2/Th17/T reg differentiation in vitro.

A caveat when interpreting the results of this study is its cross-sectional design. It is possible that the observed differences in PDC frequency and activation are caused by differences between the groups rather than the treatment per se. However, there were no differences in demographics, disease duration, or EDSS between the NTZ-treated and untreated patients. While it is possible that untreated patients are untreated due to a milder disease, other reasons for patients to be untreated include being newly diagnosed, undergoing a wash-out period between treatments, trying to become pregnant, having a fear of side effects, or fear of medical treatment. There was no evidence for milder disease in the untreated patients in Cohort I for which we had two years of prospective follow-up, but a trend towards a higher relapse-rate and shorter time to first relapse.

In conclusion, our study shows that NTZ-treated MS patients have decreased frequencies of PDCs in peripheral blood, but that the PDCs remaining in blood display a phenotype consistent with activated mature PDCs. Taken together, the data suggests that NTZ has a differential effect on the migration of activated PDCs to lymphoid tissue resulting in increased frequencies of activated PDCs in blood. Further studies are needed to determine the functional relevance of this finding. As PDCs are important regulators of both innate and adaptive immune responses, capable of inducing both immunogenic and tolerogenic responses, pharmacological manipulation of their numbers or state of activation may have significant effects on the course of the disease being treated.
